# Supplementary material for: Sigmoidally hydrochromic molecular porous crystal with rotatable dendrons
Source: Commun Chem. 2020 Aug 17;3:118. doi: 10.1038/s42004-020-00364-3 (PMC9814496; doi:10.1038/s42004-020-00364-3)
Supplement: Supplementary file 1 — Supplementary Information [file 42004_2020_364_MOESM1_ESM.pdf]

Sigmoidally hydrochromic molecular porous crystal with rotatable dendrons

Hiroshi Yamagishi *et al*.

1. Supplementary Methods

Characterization data of 1

mp 316 °C (dec.); R_f 0.10 (*n*-hexane/$CHCl_3$ 7:3, NH silica); ^{1}H NMR (400 MHz, $CDCl_3$): δ 7.28–7.32 (m, 8H), 7.41–7.46 (m, 16H), 7.69 (dd, *J* = 2.0, 8.8 Hz, 4H), 7.87 (d, *J* = 8.8 Hz, 4H), 8.18 (d, *J* = 7.6 Hz, 8H), 8.30–8.36 (m, 10H), 8.44 (d, *J* = 0.8 Hz, 2H), 10.05 (d, *J* = 8.4 Hz, 2H); ^{13}C NMR (100 MHz, $CDCl_3$): δ 109.6, 111.3, 119.8, 119.9, 120.4, 123.2, 124.3, 125.9, 125.9, 126.5, 126.5, 127.7, 128.7, 130.6, 130.8, 132.2, 134.8, 138.2, 140.6, 140.6, 141.7, 143.3; IR (ATR): ν 3044, 1614, 1599, 1485, 1466, 1450, 1335, 1314, 1281, 1231, 999, 918, 853, 812, 797 cm^{-1}; MS (FAB): *m/z* (relative intensity, %) 1271 ($[M + H]^+$, 0.2), 1270 (M^+, 0.1); HRMS (FAB): *m/z* calcd for $C_{92}H_{54}N_8$ (M^+) 1270.4471, found 1270.4495.

2. NMR Spectroscopy

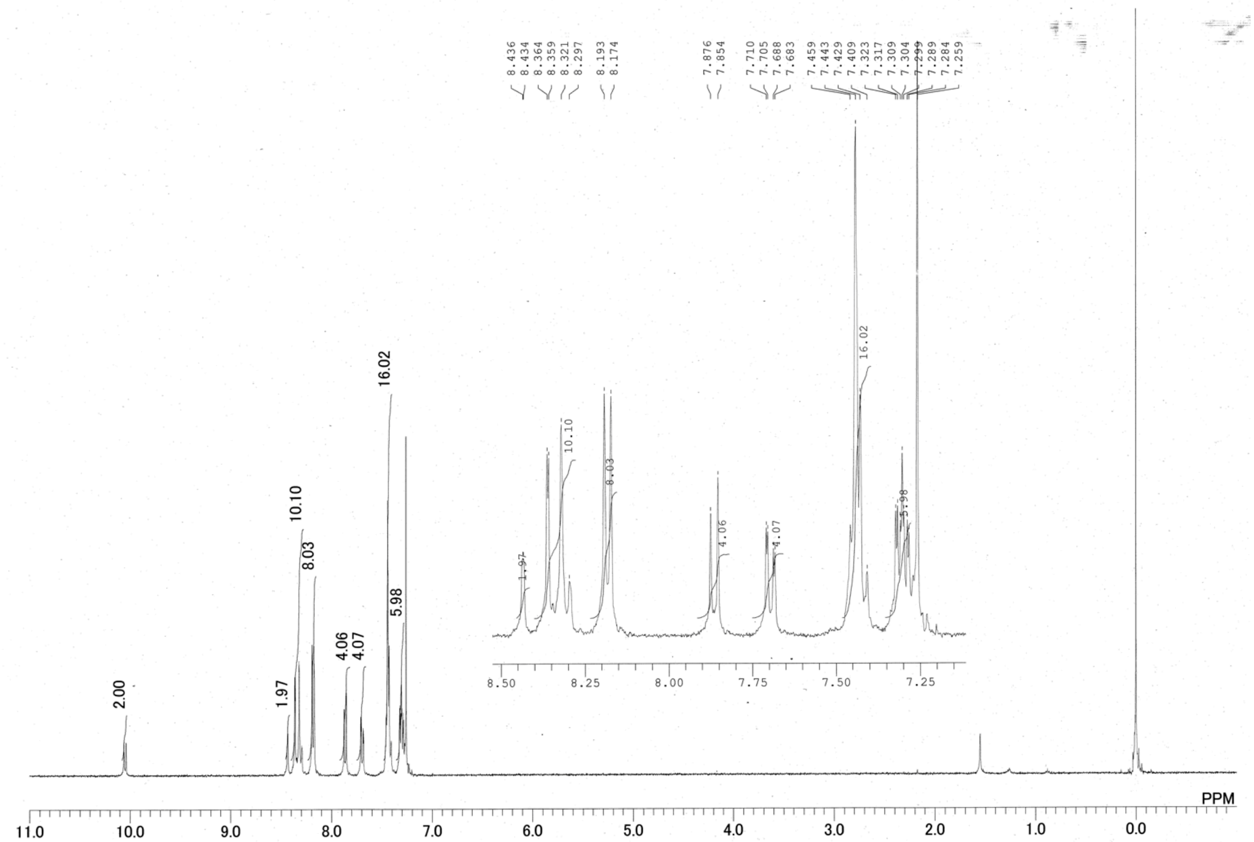

Supplementary Fig. 1. ^{1}H NMR spectrum of **1** (400 MHz, $CDCl_3$).

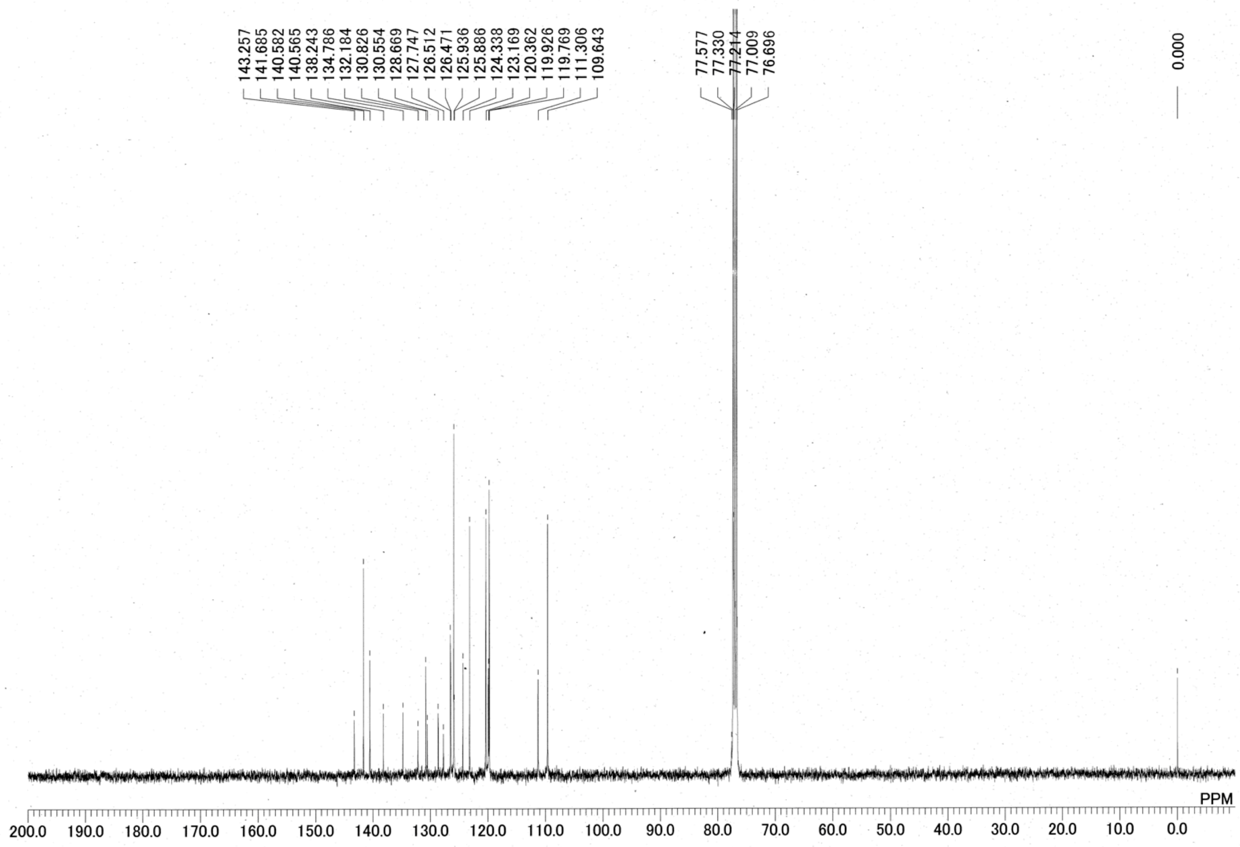

Supplementary Fig. 2. ^{13}C NMR spectrum of **1** (100 MHz, $CDCl_3$).

3. Mass Spectrometry

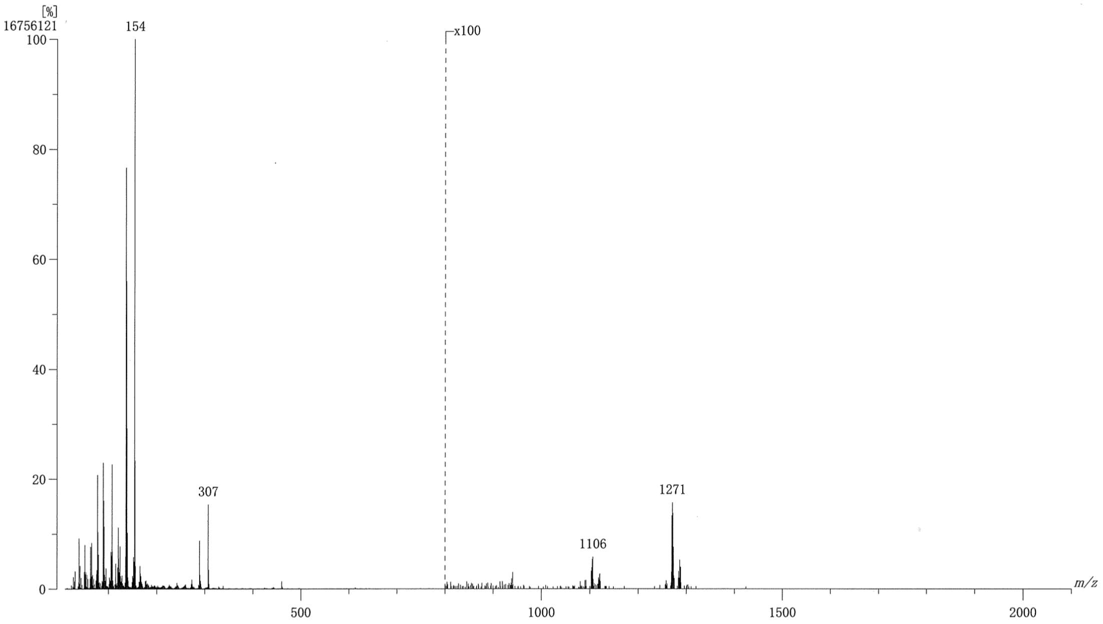

Supplementary Fig. 3. MS (FAB) spectrum of **1**.

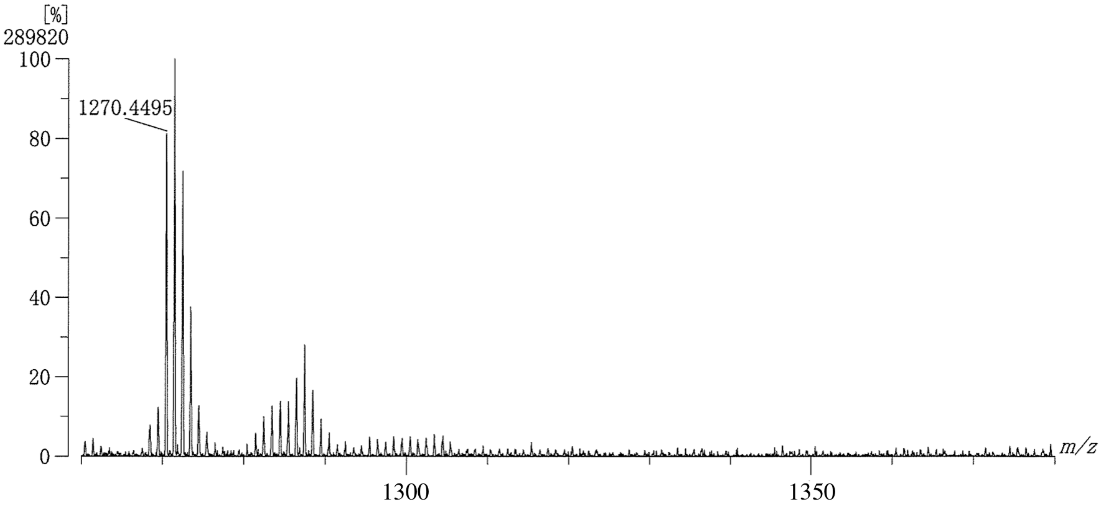

Supplementary Fig. 4. HRMS spectrum of **1**.

3. Single-Crystal X-ray Structure Analysis

Supplementary Table 1. Crystal data and structure refinement of **1**$(CHCl_3)_{1.75}$

Empirical formula	$C_{93.75}H_{55.75}Cl_{5.25}N_8$	
Formula weight	1480.32	
Temperature	93 K	
Wavelength	1.54184 Å	
Crystal system	Triclinic	
Space group	*P*-1	
Unit cell dimensions	a = 19.7687(9) Å	α = 77.410(5)°
	b = 25.7375(12) Å	β = 76.265(5)°
	c = 35.479(2) Å	γ = 88.337(4)°
Volume	17109.0(15) Å^3	
Z	8	
Density (calculated)	1.149 Mg/m^3	
Absorption coefficient	1.992 mm^{-1}	
F(000)	6108	
Crystal size	0.347 × 0.087 × 0.022 mm^3	
θ range for data collection	2.6160 to 43.017°	
Index ranges	–17≤h≤17, –22≤k≤22, –31≤l≤31	
Reflections collected	63558	
Independent reflections	24542 [*R*(int) = 0.0954]	
Absorption correction	Multi-scan	
Refinement method	Full-matrix least-squares on F^2	
Data / restraints / parameters	24542 / 6752 / 3841	
Goodness-of-fit on F^2	1.506	
Final *R* indices [I > 2σ(I)]	*R*1 = 0.1585, *wR*2 = 0.4666	
Largest diff. peak and hole	0.965 and –0.500 e Å^{-3}	
CCDC Number	2015162	

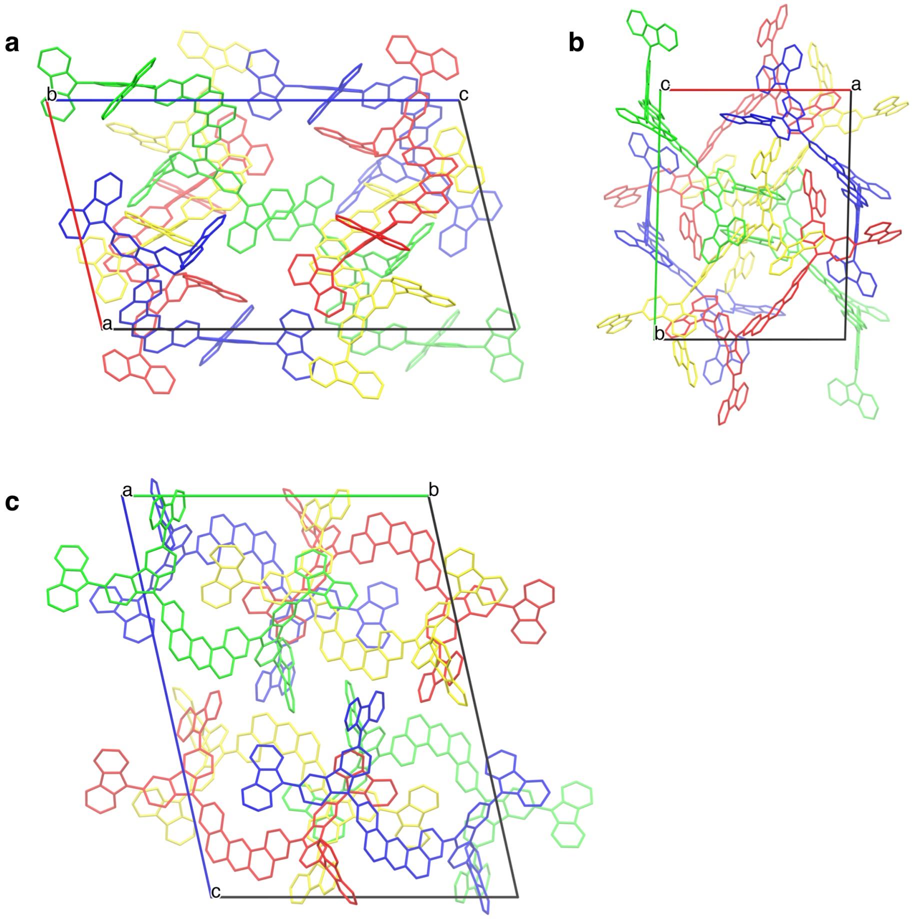

Supplementary Fig. 5. Crystal packing diagrams of **1**$(CHCl_3)_{1.75}$, viewed along the crystallographic *b*, *c*, and *a* axes (**a**, **b**, and **c**, respectively). The hydrogen atoms and guest $CHCl_3$ molecules are omitted for clarity. Symmetrically equivalent molecules are depicted in the same colour.

4. Electronic Absorption, Photoluminescence and Diffuse Reflectance Spectroscopy

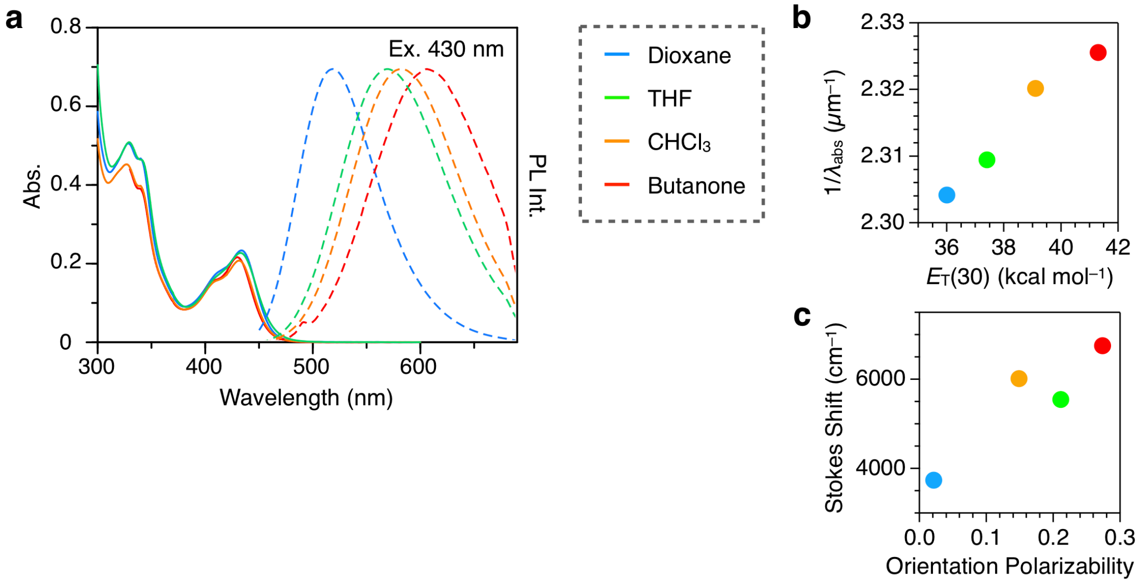

Supplementary Fig. 6. (**a**) Electronic absorption (solid curves) and photoluminescence (Ex. 430 nm, dashed curves) spectra of **1** (10 μM) in dioxane (blue curves), THF (green curves), $CHCl_3$ (orange curves) and butanone (red curves). (**b, c**) E_T30 (**b**) and Lippert-Mataga (**c**) plots of **1** in dioxane (blue circles), THF (green circles), $CHCl_3$ (orange circles) and butanone (red circles).

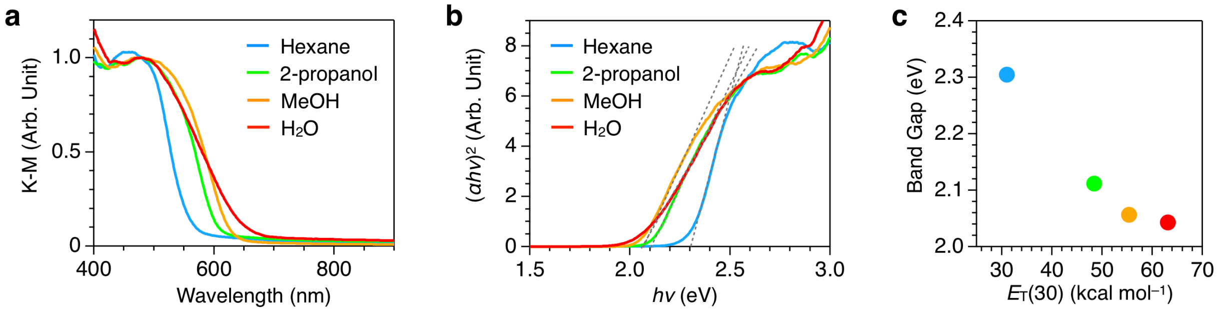

Supplementary Fig. 7. (**a**, **b**) Diffuse reflectance spectra (**a**) and Tauc plots (**b**) of **VPC-1** immerged in hexane (blue curve), 2-propanol (green curve), MeOH (orange curve), and H_2O (red curve). (**c**) The E_T30 plot of **VPC-1** immerged in hexane (blue circle), 2-propanol (green circle), MeOH (orange circle), and H_2O (red circle).

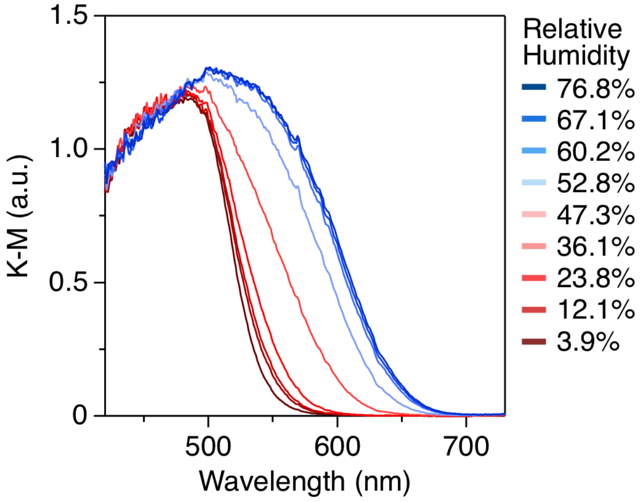

Supplementary Fig. 8. Humidity-dependent diffuse reflectance spectra of **VPC-1** upon decreasing the surrounding humidity from 76.8 to 3.9%.

5. Thermogravimetric Analysis

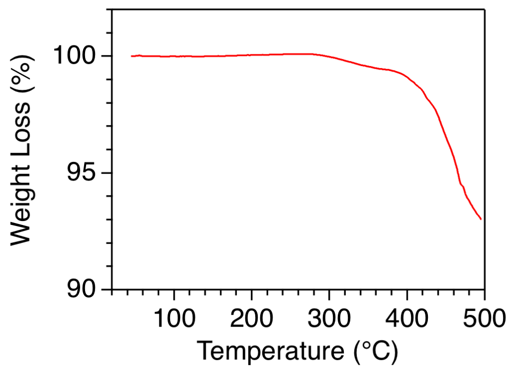

Supplementary Fig. 9. Thermogravimetric analysis of **VPC-1** upon heating at a rate of 10 °C min^{-1} under constant Ar flow. The decomposition temperature for the 5 % ($T_{5\%}$) mass loss is 461 °C.

6. Differential scanning calorimetry

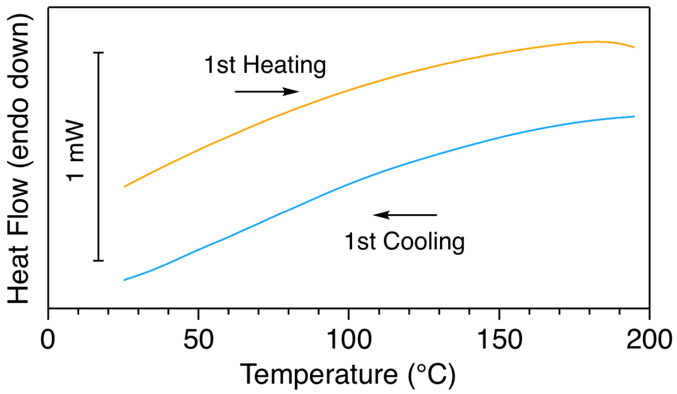

Supplementary Fig. 10. Differential scanning calorimetric profiles of **VPC-1** upon heating (orange curve) and cooling (blue curve) at a rate of 5 °C min^{-1} under constant Ar flow.

7. Powder X-ray diffractometry

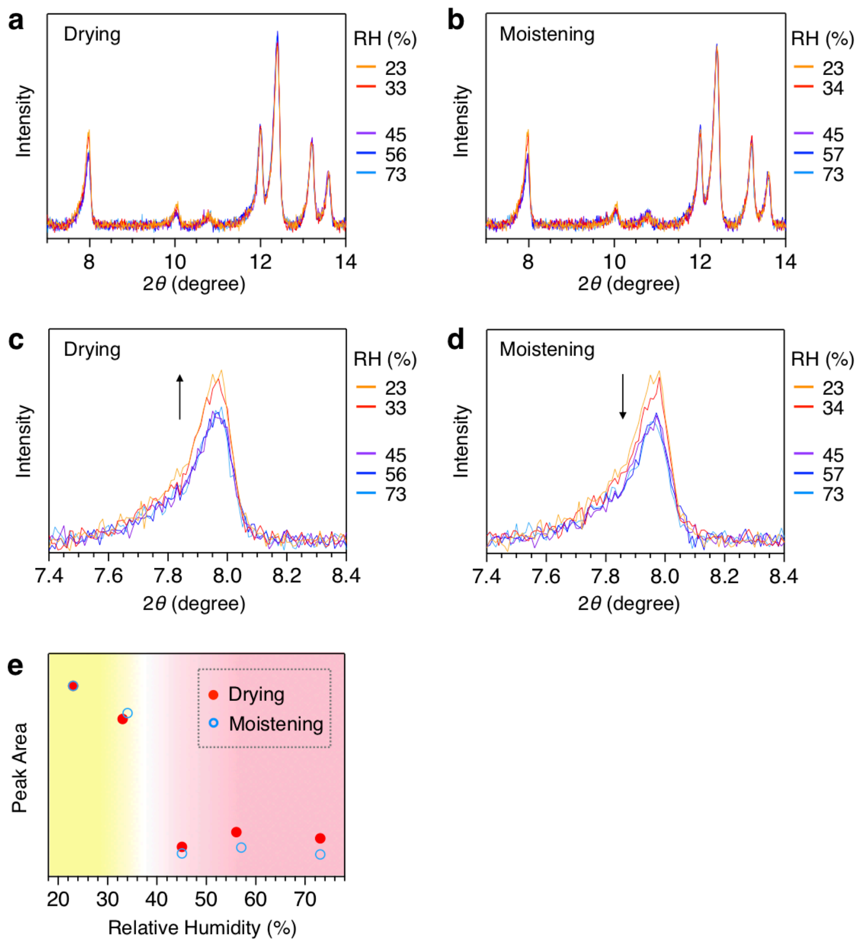

Supplementary Fig. 11. (a, b) VH-PXRD profiles of **VPC-1** upon (a) drying and (b) moistening measured under sealed chamber at constant temperature of 22.6 °C. (c, d) Magnified VH-PXRD profiles of Supplementary Fig. 11a and b. (e) A plot of the peak areas of the diffraction peak at 2θ ~ 8° against the relative humidity.

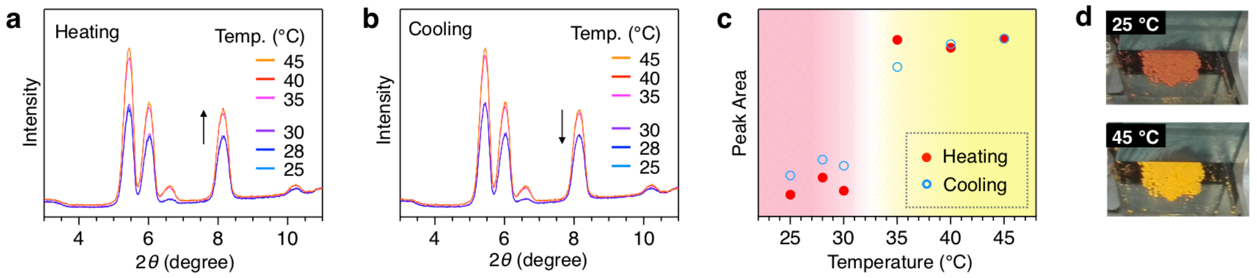

Supplementary Fig. 12. (a, b) VT-PXRD profiles of **VPC-1** upon (a) heating and (b) cooling measured under atmosphere with relative humidity of 69 %RH at 25 °C. (c) A plot of the peak areas of the diffraction peak at 2θ of 8.1° against the temperature. (d) Photographs of the powder samples of **VPC-1** at 25 (upper) and 45 °C (bottom).

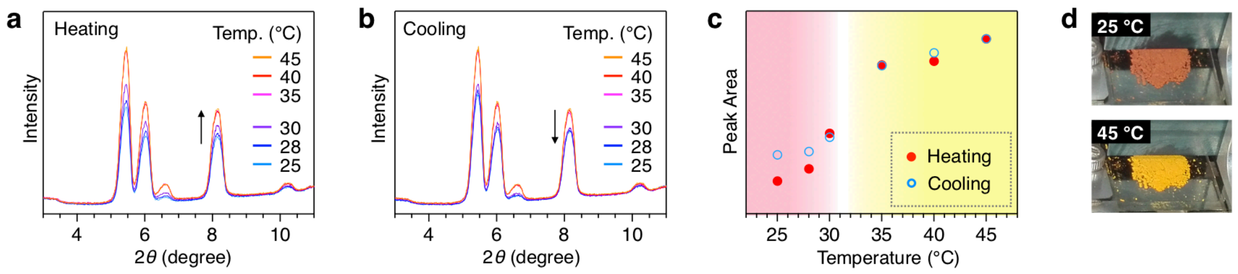

Supplementary Fig. 13. (a, b) VT-PXRD profiles of **VPC-1** upon (a) heating and (b) cooling measured under atmosphere with relative humidity of 58 %RH at 25 °C. (c) A plot of the peak areas of the diffraction peak at 2θ of 8.1° against the temperature. (d) Photographs of the powder samples of **VPC-1** at 25 (upper) and 45 °C (bottom).

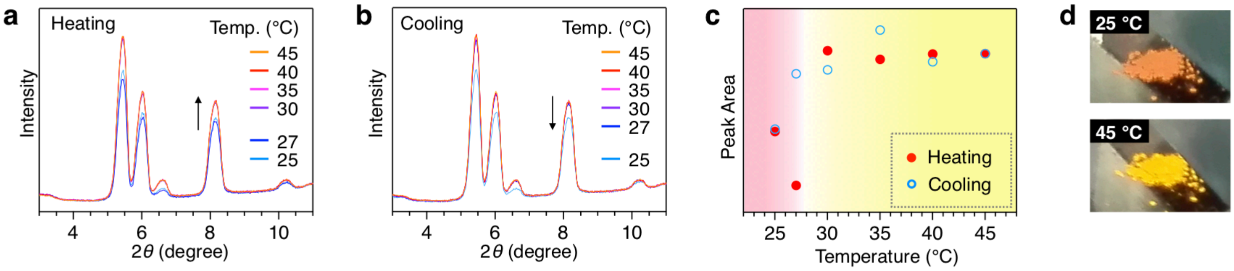

Supplementary Fig. 14. (a, b) Variable-temperature powder X-ray diffraction (VT-PXRD) profiles of **VPC-1** upon (a) heating and (b) cooling measured under atmosphere with relative humidity of 47 %RH at 25 °C. (c) A plot of the peak areas of the diffraction peak at 2θ of 8.1° against the temperature. (d) Photographs of the powder samples of **VPC-1** at 25 (upper) and 45 °C (bottom).

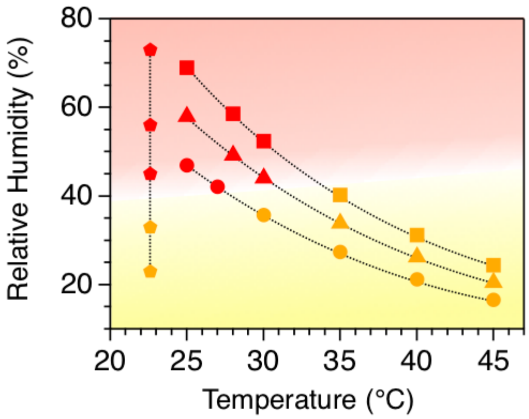

Supplementary Fig. 15. A phase diagram of **VPC-1** plotted based on the results of VH-PXRD (Supplementary Fig. 11) and VT-PXRD (Supplementary Figs. 12–14). The red and yellow markers indicate **VPC-1**$^{\mathbf{red}}$ and **VPC-1**$^{\mathbf{yellow}}$, respectively. The pentagon markers represent the VH-PXRD experiments. The circle, triangle and square markers represent the VT-PXRD experiments measured under atmosphere with relative humidity of 47, 58 and 69 % at 25 °C, respectively. The relative humidity at elevated temperature is mathematically calculated based on the reported saturation pressure of water. The plausible phase area of **VPC-1**$^{\mathbf{red}}$ and **VPC-1**$^{\mathbf{yellow}}$ is depicted as red and yellow background, respectively.

8. Pore Size Analysis

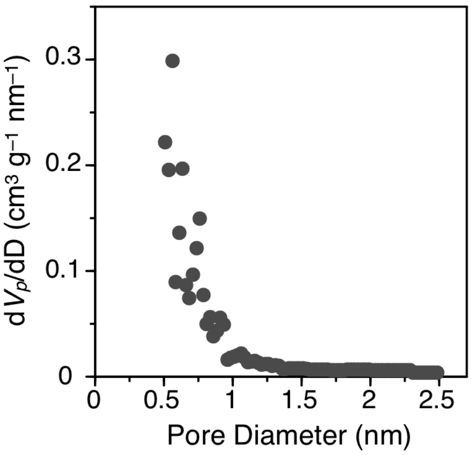

Supplementary Fig. 16. Pore size distribution of **VPC-1** calculated from its N_2 adsorption isotherm (Fig. 2f) by means of the Horvath–Kawazoe (HK) model.

9. FTIR Spectroscopy

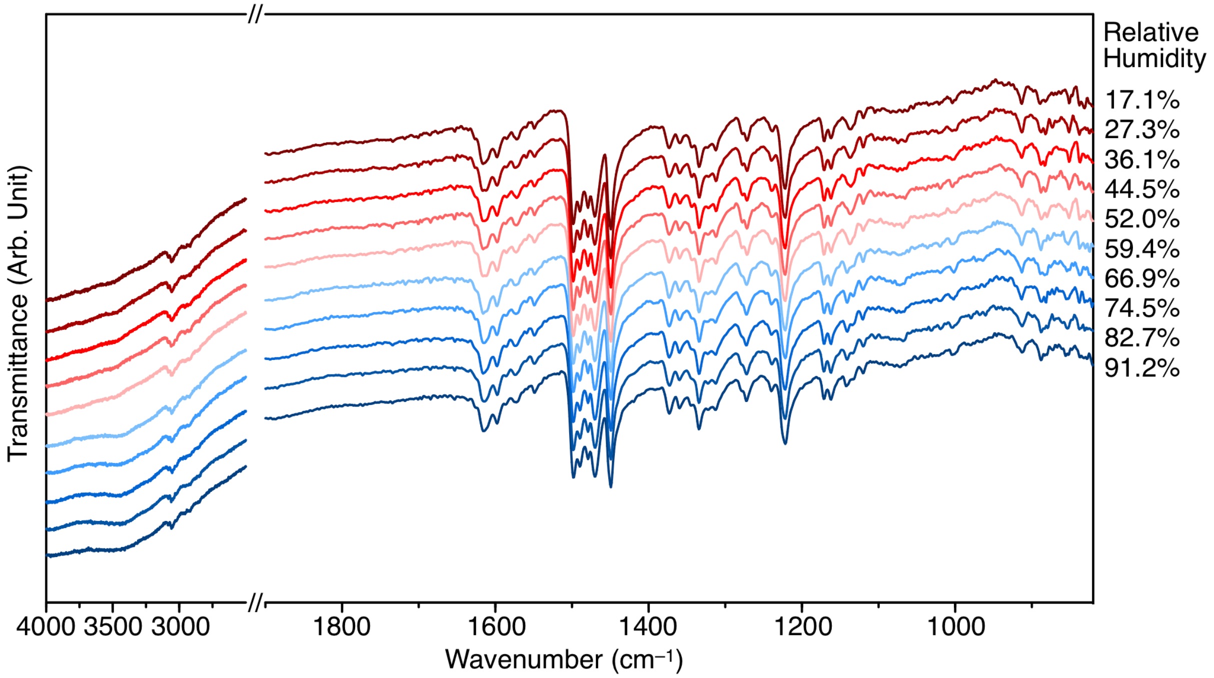

Supplementary Fig. 17. FTIR spectra of **VPC-1** measured upon decreasing the relative humidity from 91.2 to 17.1%.

10. Raman Spectroscopy

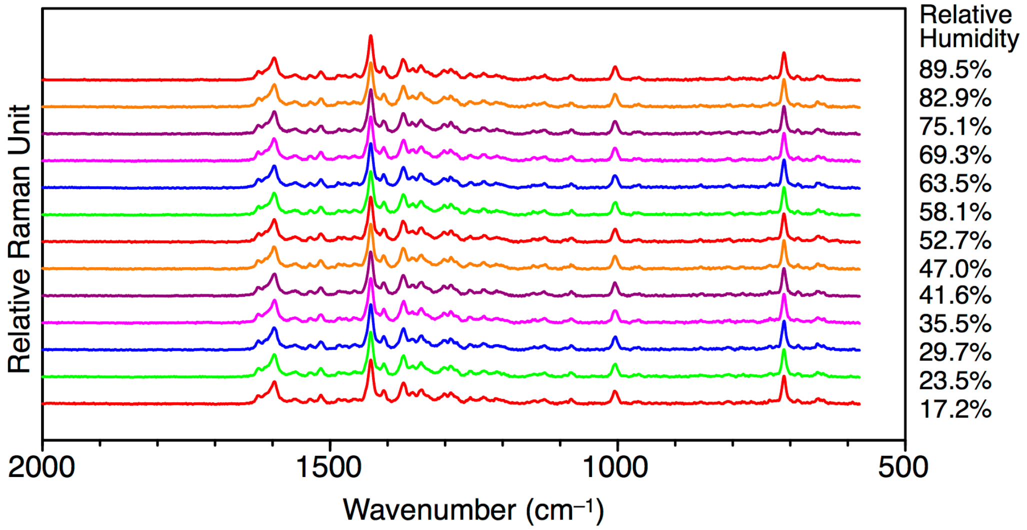

Supplementary Fig. 18. Raman spectra of **VPC-1** measured upon increasing the relative humidity from 17.2 to 89.5%.

11. DFT Calculations

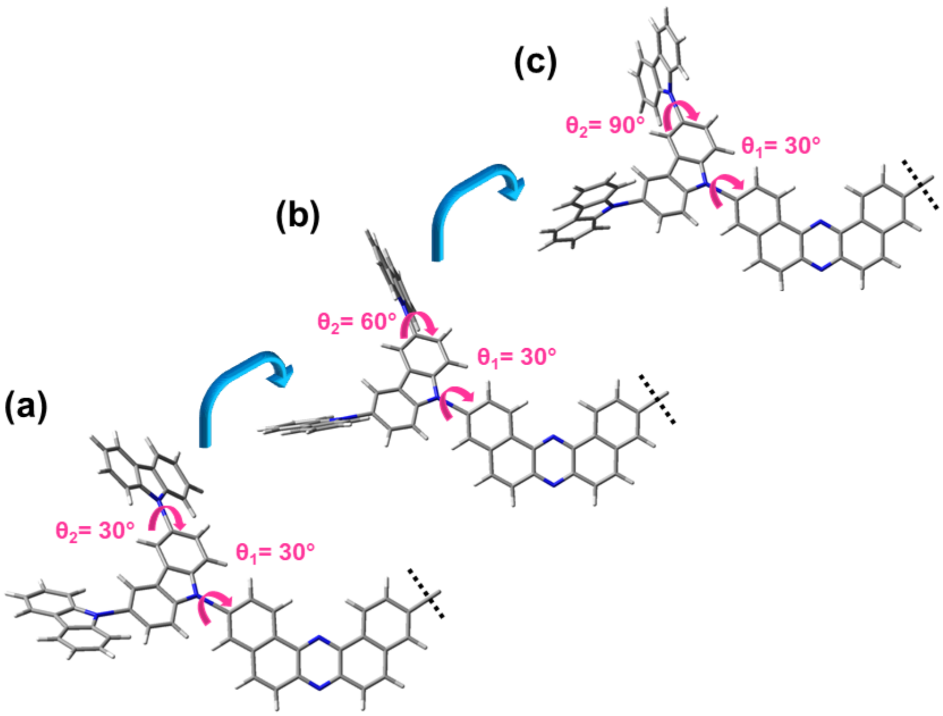

Supplementary Fig. 19. A side-view of the three **1** model systems studied theoretically: (**a**) 30/30 model, (**b**) 60/30 model and (**c**) 90/30 model, where the external Cz dihedral angles (θ_2) are twisted from 30 to 60 to 90°, respectively, whereas the internal dihedral angles (θ_1) are kept to 30°.

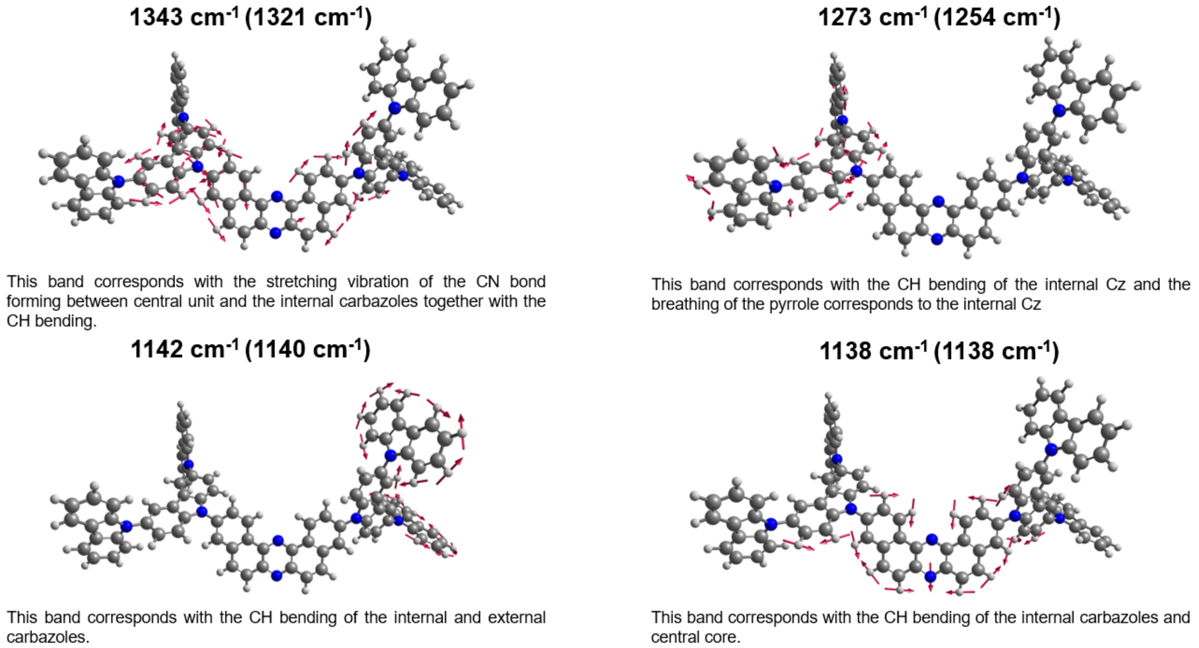

Supplementary Fig. 20. Eigenvectors (CAM-B3LYP/6-31G** level) associated to the infrared bands that are most affected by the relative humidity. The measured and theoretical (in parentheses) wavenumbers are also shown.

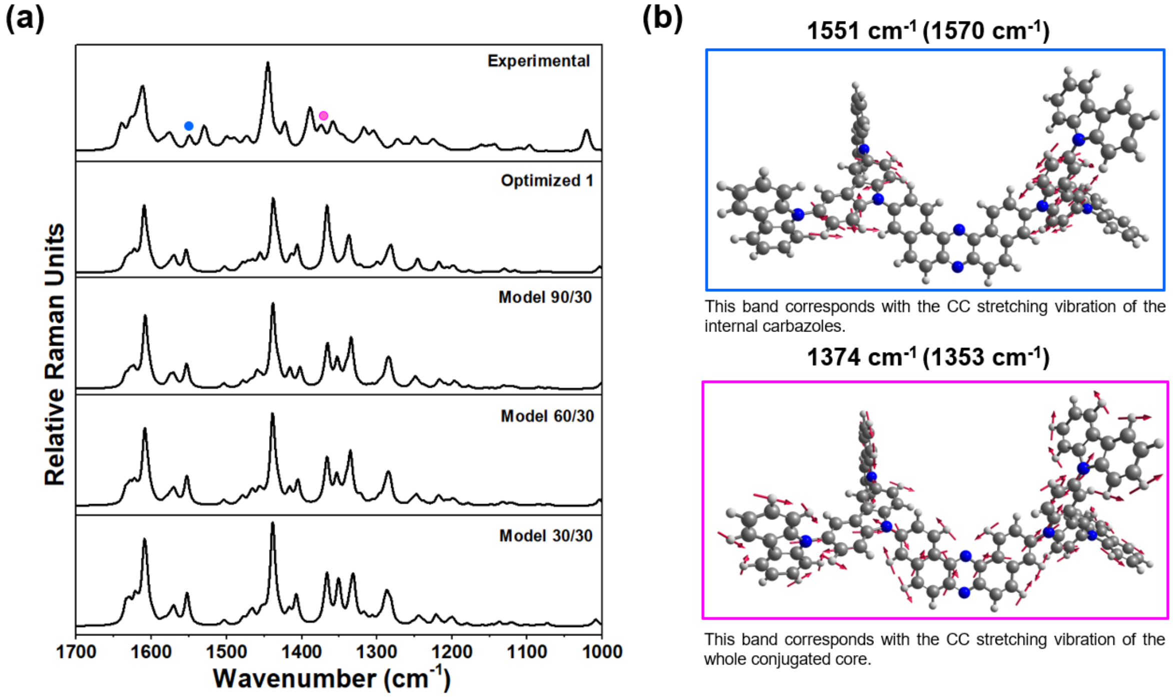

Supplementary Fig. 21. Theoretical Raman spectra (CAM-B3LYP/6-31G** level) together with the Raman experimental spectrum of **1** compound and the eigenvectors associated to the Raman bands that are most affected with the different structural conformation, respectively.

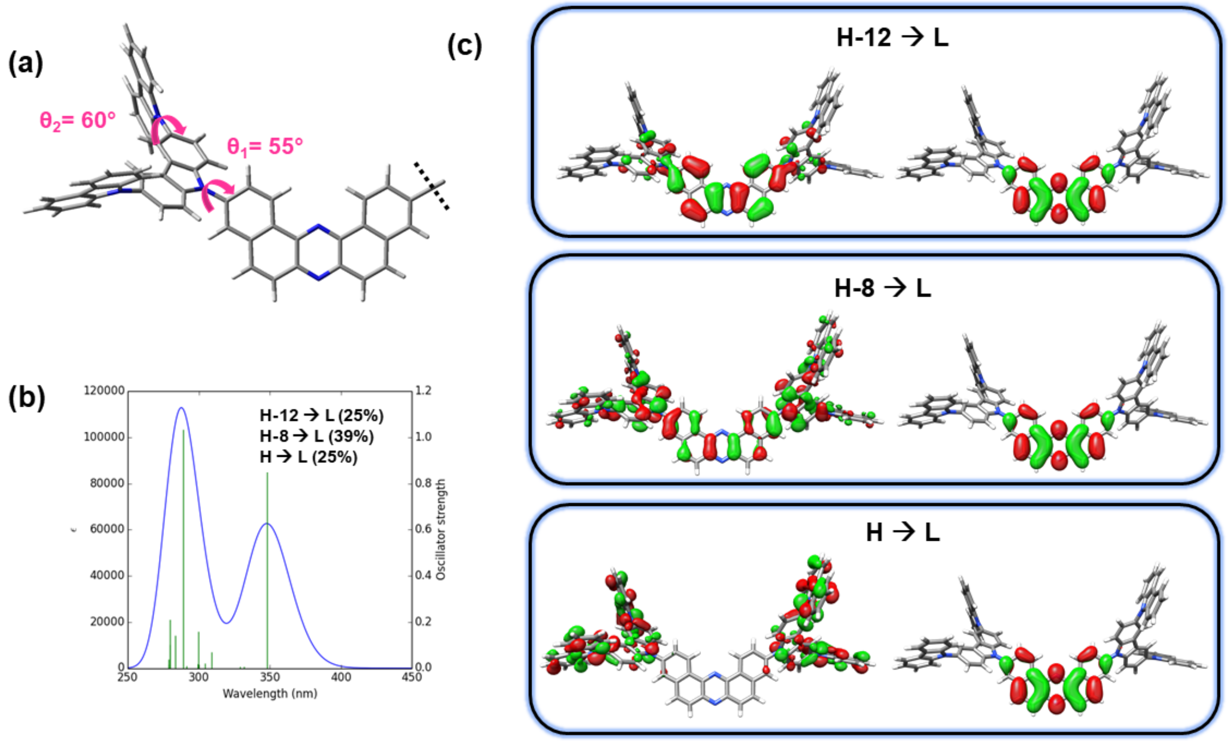

Supplementary Fig. 22. (**a**) A side-view of the optimized **1** compound where the external Cz dihedral angles (θ_2) are 60°, whereas the internal dihedral angles (θ_1) are 55º. Note that only half molecule has been displayed in the figure for a better visualization. **(b)** Simulated absorption spectra together with the excitations (wavelength vs. oscillator strength) shown as vertical bars for **1** the optimized structure as determined with TD-DFT at the CAM-B3LYP/6-31G** level (**c**). The topologies of the frontier molecular orbitals mainly involved in the lowest energy transition band are also show (H corresponds to HOMO and L corresponds to LUMO).

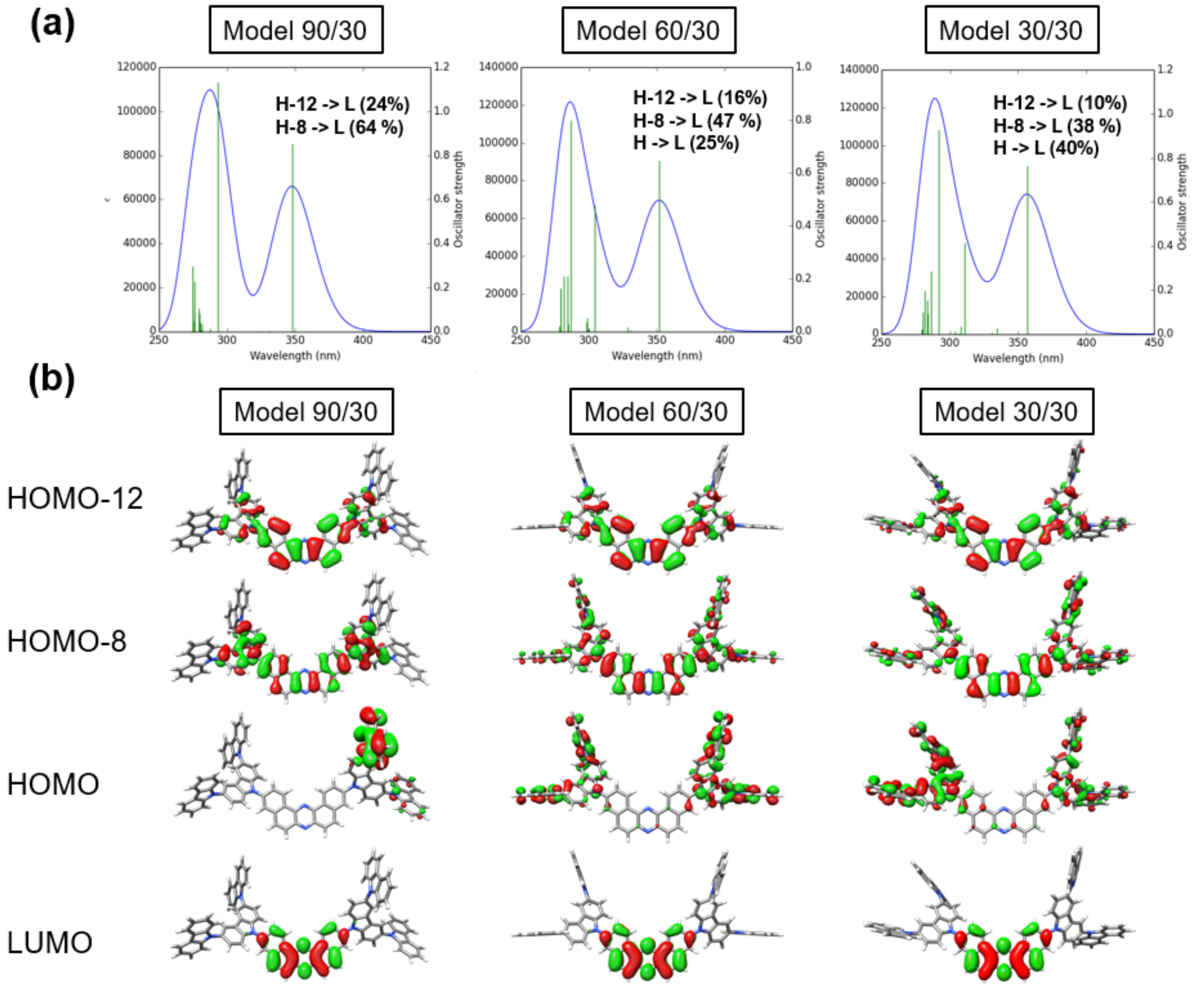

Supplementary Fig. 23. (**a**) Simulated absorption spectra together with the excitations (wavelength vs. oscillator strength) shown as vertical bars for the three **1** model systems as determined with TD-DFT at the CAM-B3LYP/6-31G** level (**b**). The topologies of the frontier molecular orbitals mainly involved in the lowest energy transition band are also shown for three **1** models.

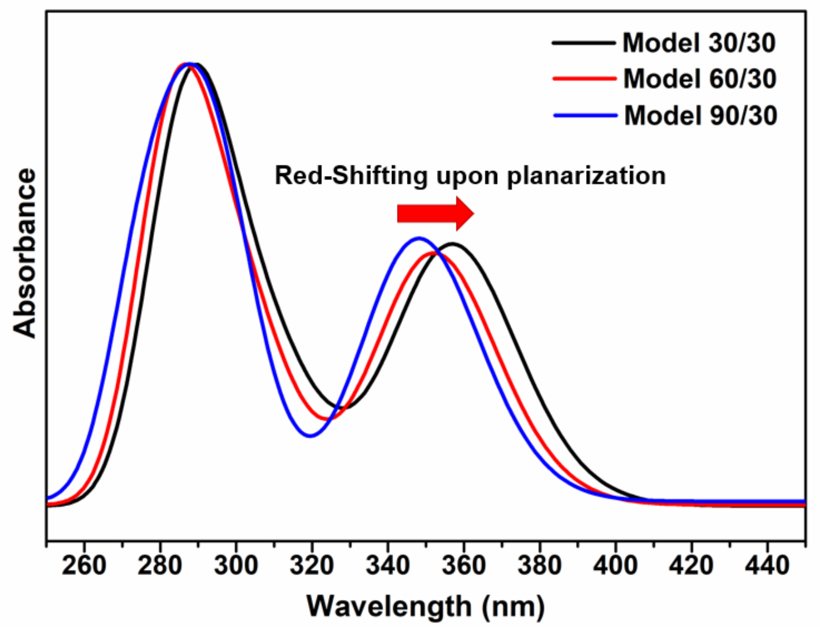

Supplementary Fig. 24. Simulated absorption spectra for the three **1** model systems as determined with TD-DFT at the CAM-B3LYP/6-31G** level.

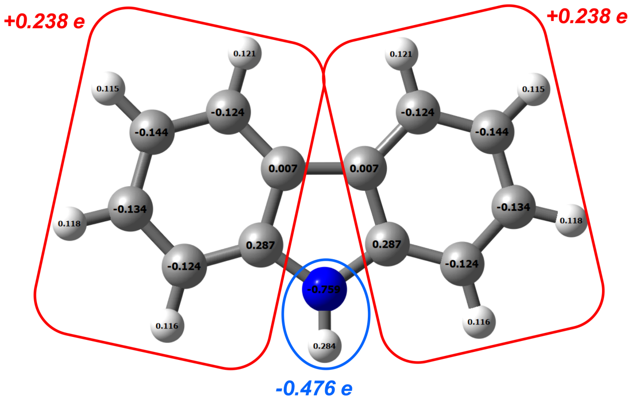

Supplementary Fig. 25. DFT-calculated atomic charge distribution of carbazole at the CAM-B3LYP/6-31G** level.
