# Supplementary material for: Sigmoidally hydrochromic molecular porous crystal with rotatable dendrons
Source: Commun Chem. 2020 Aug 17;3:118. doi: 10.1038/s42004-020-00364-3 (PMC9814496; doi:10.1038/s42004-020-00364-3)
Supplement: Supplementary file 2 — Description of Additional Supplementary Files [file 42004_2020_364_MOESM2_ESM.pdf]

Description of Additional Supplementary Files

File Name: Supplementary Data 1
Description: Crystallographic information file for compound **1**$(CHCl_3)_{1.75}$.

File Name: Supplementary Movie 1
Description: A movie recording the colour change of **VPC-1**$^{\mathbf{red}}$ from red to yellow upon drying by reducing pressure inside a glass container. The colour change was completed within 10 sec after starting the vacuuming.
